# The Nature and Treatment of Chorea
*Read, at the request of the Investigation Committee, before the West Somerset Branch


**Published:** 1884-06

**Authors:** E. Long Fox

**Affiliations:** Consulting Physician to the Bristol Royal Infirmary


THE BRISTOL

Medico-Chirurgical Journal.

JUNE, 1884.

### THE NATURE AND TREATMENT OF CHOREA.*

BY

E. LONG FOX, M.D., F.R.C.P.,

*Consulting Physician to the Bristol Royal Infirmary.*

I FEEL it a great honour to be invited to open the discussion to-night, in the presence of medical men of deservedly such high reputation.

It is not in my province now to urge the advantages of the Investigation Committee. They are probably recognised by all present. Nor in speaking of chorea will it be possible to touch on all the points on which that Committee desires information. The ages of the patients so affected, their sex, their family history with reference to this or other neuroses, the time of year, or the condition of the soil in which the disease is most prevalent—all these are considerations that are of interest, as bearing upon prophylactic measures. But these are what we may call rather the environments of the disease; and it has seemed wise to the authorities of your branch that tonight we should confine ourselves to two main questions:

* Read, at the request of the Investigation Committee, before the West Somerset Branch.

1. The nature of chorea, as evidenced by its pathology and its pathological anatomy, with special reference to its dependence on or connection with heart disease, rheumatic or otherwise.

2. The treatment of chorea—not only as a practical point of surpassing interest to us in our relation to the public, but also because, to some extent at least, the treatment sheds some light on the nature of the disease itself.

First, then, as to the nature of chorea. It is convenient to speak of it as a disease. It is really a symptom, or a series of symptoms, in relation not with the nature of the morbid change, but with its seat.

In opening up this portion of the subject, we have to deal first with the position of possible lesions.

Cases of hemichorea teach much that is valuable of the cerebral origin of the disease. In a majority of cases of ordinary chorea the symptoms are apt to begin unilaterally, but there are a certain number in which the unilateral condition is permanent. These may or may not be associated with modifications of sensation, and even with some disturbance of special sense. Hemichorea is more usually left-sided than right.

The symptoms of chorea are not only frequently excited by fright, worry, anxiety, despondency, emotion in general, but many psychical phenomena manifest themselves in the course of the disease—irritability, taciturnity, impairment of memory, incapacity for thought, general intellectual weakness, and in some cases delirium, hallucination of sight, especially at night, and even violence.

Besides these psychical phenomena, the motor symptoms are such as point to affection of the various motor centres for the face and lips, the arm and leg, that have been shewn by Hitzig and Ferrier to be in the immediate neighbourhood of the fissure of Rolando. In one form, the external muscles of the abdomen and the levator ani are involved. In the majority of cases the inco-ordination is associated with muscular weakness. Unilateral facial palsy may coexist with chorea, and disappear with it. All these points demonstrate the origin of chorea to be cerebral.

Looking, then, at *post mortem* records, the same view is borne out.

In one case of fatal hemichorea, Dr. Broadbent found no lesion, except a little lymph in the lateral ventricle. In Dr. Mabboux's case of right hemichorea with melancholia, without muscular paresis or anæsthesia, there was a blood-clot in the left lenticulo-optic region, surrounded by softening. The hæmorrhage involved the internal capsule, but not the most posterior portion of it, invading also the postero-external part of the optic thalamus and the tail of the corpus striatum. The mental symptoms were accounted for by chronic fronto-parietal meningo-encephalitis. The cerebral origin of hemichorea is well seen in some cases examined after death by Charcot. Here hemichorea followed hemiplegia; and there was not only anæsthesia of the skin on the hemiplegic side, but on the paralysed side the organs of special sense were more or less involved. The lesion was a cicatrix of the posterior end of the optic thalamus and the nucleus caudatus, and the hindermost portion of the foot of the corona radiata. In two cases the anterior corpora quadrigemina were affected. The changes which concerned the chorea were located in motor fibres, situated in front and to the side of those parts of the corona radiata which preside over the conduction of sensitive impressions.

Hemichorea and anæsthesia are also found preceding hemiplegia, the lesion involving the posterior half of the optic thalamus. Tumour in this position may cause hemichorea, which may or may not be followed by hemiplegia.

Two cases of fatal hemichorea have lately been published in the "*Archives für Psychiatrie.*" The one was a case of post-hemiplegic left hemichorea ; and the lesions found were two small separate hæmorrhagic spots in the right thalamus. A spot of softening in the right occipital lobe. The left cerebral hemisphere was healthy. A spot of softening in the left cerebellar hemisphere. Hæmorrhage in left half of the pons between the trigeminus nerve on the upper end of the pons. In the deeper part of this organ, and of the medulla oblongata in both pyramids, there was a moderate amount of hyperplasia of the interstitial tissue, more on the right than on the left side. In the cord there was degeneration of both posterior columns throughout their whole extent, especially on the left side. In the lower dorsal region there was a fresh myelitis, especially of the posterior columns.

The other case, also of left hemichorea, shewed aneurism of the ascending aorta, chronic entarteritis, cystitis, and œdema of the lungs. The cerebral dura and pia mater were more or less thickened: the upper surface of the brain discoloured. There was ecchymosis of the upper cortex, and a granular condition of the ependyma of the fourth ventricle. The spinal dura and pia mater not quite healthy. The substance of the cord firm. On minute examination, in the right hemisphere of the brain the vessels were blocked with cells, and small extravasations were seen, particularly in the cerebral convolutions and in the paracentral lobule. Numerous spider cells in the upper part of the white substance.

Commencing spots of softening in the upper part of the right half of the pons, near the right crus cerebelli ad pontem. Secondary descending degeneration of the right pyramid through the pons and medulla. In the cord there was degeneration of both postero-lateral columns, chiefly of the left: moderate degeneration of both columns of Goll, especially in the dorsal region, more slight in the cervical.

The following notes are of three fatal cases of my own, in which a minute examination was made.

1. Amelia H., 17. Choreic three weeks. Much jactitation of both arms and both legs, and of facial muscles. Difficulty in protrusion of tongue and in speech. No cardiac bruit. Slight opisthotones. Mind rather weak. She became suddenly comatose after much violent movement. Right pupil much dilated, left contracted. Death.

Some clots of blood were found lying outside the brain on the right side in the arachnoid space. It was thought possible that this blood had come from a small orifice in the right lateral sinus. Small capillary emboli were found in the arteries of the corpora striata.

A fringe of bead-like excrescences of lymph, that could be detached by the finger, were found round the auricular boundary of the mitral orifice.

2. Emily S., 17. Rheumatic history. Systolic bruit at base. Bilateral movements. Difficulty of articulation. Is confused and somewhat delirious, but had had some diplopia and confusion for several months before the chorea. No catamenia for four months. No pregnancy. Was improving at the end of three weeks, but suddenly manifested dyspnœa, and died in three minutes.

Several large bed-sores. Brain a little soft everywhere, especially in the central white matter. Nothing abnormal to be discovered by the naked eye, except that on the convexity a little grey matter came away here and there with the pia mater. A little thickening of vessels of the medulla oblongata, and some increase of white corpuscles in them. A minute vegetation on each corpus arantii of the aortic valve. The auricular border of the mitral valve rather thickened, not beaded. A firm clot in right ventricle, which extended some distance into the pulmonary artery.

3. John T., 17. Bilateral violent jactitation. Hallucinations of sight. Morbid movements ceased on the ninth day, but he rambled on in delirium for seventeen days, and died of exhaustion.

Dura mater very adherent along superior longitudinal sinus. Consistence of brain firm. The whole brain very full of blood. The corpora striata and optic thalami perfectly healthy to the naked eye. Pons rather injected. Olivary bodies seemed fibrous. The whole of the medulla oblongata rather tough. Microscopically, emboli were seen in the vessels of the corpora striata, and the vessels and perivascular spaces were dilated. Spots of sclerosis in the olivary bodies. Some beading of the auricular edge of the mitral valve. Both lungs profusely studded with small indurations, varying in size from a pin's head to a small nut. Each of these indurations seemed to be an infarctus, formed apparently by a thrombus in an ultimate branch of the pulmonary artery, behind which in some cases the vessel had given way and caused hæmorrhage. Some of these spots were on the road towards disintegration and abscess. Spleen small, and contained an infarctus.

Besides the *post mortem* appearances that prove the cerebral origin of chorea, Dr. Reynolds long ago formulated certain reasons against the spinal cord being primarily the seat of the disease. He said: "Choreic spasm of this incessantly repeated character is not a phenomenon of persistent spinal irritation. (Tonic spasm is the mark of such a condition)."

The movements, unless very severe, and even then to some extent, can generally be somewhat controlled by the will, and it is certain that the purely asensuous reflections are not amenable to volition; at all events to the same extent.

The spasmodic contractions cease during sleep, whereas the phenomena of excito-motory character are increased by the removal of volition.

The direction of attention to some other object likewise diminishes the intensity of choreic movement.

The special occasions of increase or of induction of choreic movements are the attempts at volitional action and emotional changes.

To these dicta may be added the fact, that in cases of hemichorea with anæsthesia, the motor and semory impairment are on the same side.

Other lesions have been found, varying somewhat in position, both in the brain and spinal cord.

Blocking of arteries (posterior and middle cerebral) and consequent softening, with hyperæmia of the surface of the brain, and swelling and softening of the anterior columns of the cord.

"Inflammatory changes of the cells of the cortex and basal ganglia, as also of neuroglia and Deiter's cells in the cord."—*Meynert.*

"Inflammatory changes of spinal cells and neuroglia, and thickening of the ependyma of the central canal."—*Elischer.*

"Dilatation of vessels, hæmorrhages, or sclerosis of the corpora striata, and in the cord a similar condition of the central portion of each lateral mass of grey matter, comprising the root of each posterior horn."—*Dickenson.*

Plugging of anterior and antero-lateral arteries in the cord.

Ross thinks the corpora striata and optic thalami the main centres of lesion in chorea, and perhaps, too, the cortical convolutions near the corpora striata; and that the condition of the nervous matter is rather that of anæmia than of hyperæmia.

Aitken found the specific gravity of the corpus striatum and optic thalamus very much diminished in proportion to the specific gravity of other parts of the brain.

There are instances, too, of congenital chorea from incomplete development. Charcot mentions hemichorea in children caused by partial atrophy of brain. I have met with two cases of congenital chorea, in both of which the arrest of development seemed to be due to maternal fright during pregnancy.

1. Henry S., 13. Has had chorea from his birth. His mother went about seven and a half months with him. She was frightened when about two months pregnant. He has always been as bad as, if not worse than, at present. He eats, drinks, and sleeps well. Lately has learned to say a few words, but previously could not speak at all; but he could hum tunes in an indistinct way and make various noises. Cannot stand, nor hold anything firmly. He is in constant motion, except when asleep. No family history of rheumatism. No heart disease. Had fits about three years old; not since. Is quite intelligent.

Under arsenic and good food, and a good deal of trouble in teaching him to move to a tune, he learned to stand upright, and to walk with the help of one hand of another person.

2. Master E., 4. Was born prematurely. The mother, when pregnant, was frightened by a snake. No nystagmus. Sees and hears well. Has never stood. Sensation good. Reflexes good. Left hand the strongest; both hands choreic. He cannot feed himself. Tendency to contracture of left leg, but it can be extended. Right leg much contracted, and all attempt at moving it causes pain. This contraction is worse during sleep.

I thought this child was suffering from malformation of the anterior portion of the internal capsules, and subsequent lateral sclerosis.

Another theory has been put forward by Elischer, that chorea depends on diffuse irritative processes of the peripheral nerves of the brain and cord, such as proliferation of nuclei in the connective tissue of peripheral nerves and along the vessels of the cord. This theory is interesting as bearing upon the so-called reflex chorea of pregnancy.

Besides the psychical, the sensory, and the ordinary motor phenomena of chorea, we meet sometimes with an irregularity of heart in such patients, doubtless of a choreic origin. Pigmentation of the nuclei of vagi has been found in one case. In most, however, of such examples of chorea of the heart, the irregularity depends on imperfect nutrition of the cardiac ganglia of the sympathetic, and is, perhaps, one of the few instances of the sympathetic being involved in this disease.

Dr. Henry Chapin, *New York Medical Record*, speaks of chorea of the respiratory muscles and larynx. In one of his cases there was a barking noise during expiration.

There was sudden spasm of the pharyngeal muscles; the epiglottis was raised; the adductors quickly contracted the glottis. In the second case there was spasm of expiration, with or without the above conditions. The parts most usually implicated therefore in chorea are the basal ganglia and the cortex in their immediate neighbourhood; and the various regions of the internal capsule, the part of this organ that is involved determining the co-existence or not of anæsthesia. By separate lesions, moreover, various portions of the cord are diseased. The crossed pyramidal columns, the seat of descending lateral sclerosis, are notably free.

What, then, is the nature of the lesion implicating these localized areas? Dr. Broadbent sums up the matter by saying: "Embolism; other disturbances of nutrition in the ganglia; peripheral influences which arrest the reflex process; direct lesion of the ganglia from fright; mechanical lesion of the corpora striata, the optic thalami, and their vicinity, may each and all give rise to the phænomena of chorea."

To take embolism first. Out of a good many cases of chorea treated by myself, I have only notes of ninety-six on the question of heart disease. I have only been able to recognize heart disease by physical examination in seventeen of these—a less proportion than the 27 per cent. obtained by the Committee—although a large proportion of my cases had either been rheumatic or owned a rheumatic family history. Out of five autopsies, however, heart disease was found in three. Dr. Sturges says, what we shall all probably agree to, that vegetations, especially of the mitral valve, or pericarditis, are found in the majority of fatal cases. He thinks, however, that this condition rarely contributes to the fatal issue. It is found equally in those cases that die with and those that die of chorea; and in some marked cases the heart is found healthy.

The position of the beading of the mitral valve accounts for the frequency with which there is no morbid sound heard. These vegetations are generally attached to the auricular surface of the mitral valve, neither impeding the free action of the valve, nor materially obstructing the free flow of the blood towards the ventricle.

The so-called reflex chorea of pregnancy is so common in young women that Dr. Wilks never sees chorea in young adult women without making special investigations as to the existence of the pregnant state. Considering how markedly the brain, and especially the sympathetic in the brain, is excited by all kinds of disturbances in the pelvic organs, it would be by no means strange that under such circumstances chorea would be purely reflex. It is easily conceivable that from pelvic irritation the nutrition of the basal ganglia may be modified, and choreic phenomena result, as occurs in those numerous instances of chorea minor, which recover under almost all circumstances. But Elischer observed in a pregnant woman, who had suffered from chorea, nuclear proliferation and hyperplasia of the connective tissue in the corpus striatum, thickening of the tunica adventitia of the small vessels, the same changes in the tunica intima of the vessels of the lenticular nucleus, and division of the nuclei of the nerve-cells in the claustrum. The spinal cord presented thickening and nuclear proliferation in the walls of the vessels, inflammatory changes in the epithelium of the central canal, and nuclear proliferation in the connective tissue round the nerve-cells of the grey matter; the latter presented a dull appearance, were destitute of nuclei, and filled with pigment; the white substance was hyperæmic; the lateral and posterior columns contained a fibrillary tissue strewn with nuclei. In the peripheral nerves the fibres were diminished in number; a large amount of connective tissue was interposed between them; there were small hæmorrhages in the interstices of this tissue.

Other observers also consider the chorea of pregnancy to be not reflex, but a propagation to the cord and brain of inflammatory conditions of the peripheral nerves.

The ephemeral duration of this form of chorea, as a rule, leads one to believe that these lesions are not common. In one of my own cases the phenomena seemed to depend on the presence of a lumbricus in the intestines, and to subside on the expulsion of the worm; showing, at any rate, that reflex chorea is possible. It is at this point, among others, that chorea may touch hysteria, the phænomena of which so often own, as their proximate cause, intestinal or pelvic irritation.

It is chiefly in connection with pelvic irritation that we see the best examples of rhythmical chorea. In the most marked case I ever met with, the arms and legs moved alternately with great force for some days together. The patient nearly sank from exhaustion. Pressure over the ovaries may cause temporary cessation of the movements. It is not to be confounded with chorea minor, but belongs to the systematic chorea of Sée, in which the involuntary movements are, so to speak, co-ordinated. It is, perhaps, more nearly allied to hysteria than to chorea.

Another form of chorea is seen in old people. There is no cure for it, but it does not destroy life. There are two types of this: one, in which the involuntary movements, increased by attempts at voluntary motion, cease during sleep; and another, in which even during sleep there is no cessation of the movements. It is an emotional disease in the sense that the phenomena are intensified by emotion. It has no connection with rheumatism, and is often associated with dementia. The disease differs from senile trembling, in which the head is first troubled; as also from rhythmical spasm of the muscles of the neck. But it is probably nearly related to, or at least would form part of, the same group as paralysis agitans, and multiple sclerosis of the cerebro-spinal centres.

Charcot would place athetosis among the choreas, a condition in which there is almost constant movement of the fingers or toes; forced attitudes, which are exaggerated by voluntary movements, and which make it impossible for a patient to seize or hold an object.

But when all is said on the side of pathological anatomy, there remain a very large number of cases, with symptoms of greater or less intensity, in which the course of the disease necessitates the view that they are suffering from no permanent lesion. Such examples of chorea minor—of enormous importance to us as practitioners, because so common—used to be termed instances of functional disease. The term is not open to much objection if its meaning is explained; but the public are apt to understand the word functional as carrying with it the meaning of an absence of all lesion. That is assuredly inaccurate. That in the exquisitely-arranged system of the human body any derangement of the normal movements could take place without a causative derangement in the parts that rule such movements, would be an idea as paradoxical as that any movement could take place without destruction of tissue.

Functional disease means a condition that depends upon lesions that cannot be recognized easily, or at all *post mortem;* that are in their nature transitory; that are concerned specially with alterations of the relation between tissue and blood supply, either with difficulties in osmosis, or with changes in the character, or with variations in the quantity of the blood. And although any such interference with healthy laws would in time cause some tissue change; although probably such tissue change occurs, as by way of pigmentation of nerve-cells or partial atrophy; yet, when under good hygienic conditions—especially in youth, the period of rapid repair—the blood supply is improved, these tissue changes are recovered from, the morbid phenomena depending on them vanish; and if an opportunity were afforded of examination of the organ, nothing abnormal would be found—not because it has not been present, but because the morbid state has been perfectly recovered from.

With this limitation the expression "functional disease" may hold; and it is precisely this that is met with in the majority of cases of chorea minor. Look for a moment at the anatomy of the vessels that supply the basal ganglia. The branches of the middle cerebral artery that supply the corpus striatum are as different as possible from those that supply the convolutions of the cortex. Anastomosis between these latter are by no means free, but the small straight branches from the silvian artery that supply the corpus striatum are absolutely terminal arteries. There is a complete absence of anastomosis between them and other arteries. It follows, therefore, that a coarse lesion, like an embolus, in one of them, leads to destruction of tissue in the region beyond as a certainty; and that there is no possibility of the part being nourished by collateral circulation. The symptoms of such a lesion are so definite, that they could not escape observation. When, therefore, we see so large a majority of cases of chorea recover, and recover without any muscular weakness, and without any incoordination, we may be certain that we have not had to deal with embolism of one of the terminal branches of the silvian artery.

Disturbance of the nutrition of the ganglia from whatever cause, many of these disturbances such as may be recovered from, afford the only possible explanation; and the truth of this statement is shown by the effects of rational treatment.

It would be interesting to follow out the modus operandi of the various exciting causes of chorea in this relation; of fright, for instance, mental worry, insufficient food, or the rheumatic diathesis. It is constantly said that the sympathetic is unaffected in chorea. How can that be, when it is the only channel of conduction to the branches of the silvian artery and others of the influence of emotion, so potent an element in the causation of the disease? The choreic phenomena do not manifest themselves as the immediate sequence of fright, &c.; but when the influence of emotion has been brought to bear on the nutrition of certain regions, a condition not immediately induced, the motor symptoms follow as a direct consequence of the impaired nutrition. The predominance of the female sex so affected is a proof of the influence of emotion. More gradually, and therefore less commonly recognised, are the choreic jactitations the result of insufficient food. The causation is twofold, mainly, of course, the poverty of the blood; but indirectly the deficient vis a tergo, from the inability of a badly nourished and debilitated heart to supply the cerebro-spinal centres.

And then once more the rheumatic diathesis. Has this any influence over and above the direct lesion of emboli from the valves, or the reflex effect of pericarditis on the cerebral vessels? The comparative rarity of fatal results renders it difficult to answer this question, as disease of the heart has frequently been found in fatal cases where no physical signs have been observed during life. The relationship between the two conditions may vary also in different countries. According to observations made at Prague by Steiner, among 252 cases of chorea, only four appeared in the course of acute articular rheumatism, a proportion that differs very much from observations in England and France. But even in these countries there is a certain difference observable, as among 128 children affected with chorea, Sée found 64 cases of acute articular rheumatism; whilst so far our English observations go to show that, although the relationship between rheumatism and chorea is at least as close—indeed, according to Watson, even closer—yet the form of rheumatism is neither violent nor obstinate: the more acute, severe, and extensive the articular affection, and the more it is complicated, either in the beginning or at a later period, with cardiac affections, the slighter will be the choreic manifestations. On this point we await with interest the results of the vast material that is being gradually accumulated by the Investigation Committee; but if it is the case that the later stages and the slighter forms of rheumatism are those most usually associated with chorea, may it not be that (emboli and pericarditis being excluded) the more rapid circulation of a highly feverish state is less likely to lead to phenomena, which own as their cause an impaired nutrition of the nervous ganglia, such as might more readily result from a feeble blood-supply, from a nutritive fluid deficient in quality, and one perhaps all the more fitted to allow thrombosis of the smaller vessels.

One of the main difficulties in the explanation of the connection between the morbid anatomy and the symptoms of chorea is, the ignorance in which we still are as to the exact position of co-ordinating centres in the brain. The appearances above cited are enough to prove that the brain is the chief organ implicated, and *that* in various regions. Some of the cases on record prove, too, that the cord is similarly affected, and mainly in its posterior regions. But in so far as we may apply to the human frame experiments performed on the lower animals, there are some facts that show how large a share the spinal cord in them takes in the production of the choreic symptoms. In an animal which suffered from general chorea, Chauveau divided the cord near its junction with the brain, and the movements persisted for several hours, until death, without being lessened in intensity or otherwise modified. The choreic movements were therefore not dependent upon the brain or cerebellum. In two other choreic dogs, the same experiment did not check the movements, which only diminished after section of the dorsal cord, and coincidently with the arrest of the cardiac pulsations. Legros and Onimus found that upon opening the spinal canal, and irritating the posterior columns of the cord with a scalpel, the choreic movements were enormously intensified. From variations in their experiments they concluded that the site of chorea in dogs is either in the nerve cells of the posterior horns, or in the fibres which connect them with the motor cells.

From the whole consideration of the pathology of the subject, we may conclude that chorea depends on imperfect nutrition of various centres of the brain and spinal cord; that this impaired nutrition may be—

1. Congenital; frequently depending on mental shock to the mother during pregancy.
2. Due to rheumatism, either from emboli, from thrombus, from a disordered quality of the blood, or from reflex influence of pericarditis.
3. Due to inflammatory changes, not necessarily rheumatic.
4. Due to emotional causes, acting through the sympathetic.
5. Due to insufficient food and debased hygienic conditions.
6. Due to reflex influence, especially pelvic and abdominal irritation.

Whilst as to the regions chiefly involved, they are—

1. The regions supplied by the short terminal silvian arteries.
2. The optic thalami.
3. The internal capsule, sometimes the anterior portion, sometimes the posterior, and then associated with anæsthesia, and occasionally with some affection of sight.
4. The motor area of the cortex.
5. The corpora quadrigemina.
6. The cerebellum.
7. The pons and medulla oblongata.
8. Sometimes the lateral and posterior columns of the cord; more frequently the posterior cornua and their connection with the motor cells.
9. Sometimes peripheral neuritis.

---

As to treatment. It is evident from the study of the pathology and definite lesions of chorea, that, whilst one general principle, that of improving an impaired nutrition of nervous ganglia or groups of nerve cells, will underlie all variations of treatment, yet differences will arise according to the intensity of the symptoms, the special conditions that may threaten the fatal event, the nature of the positive lesion, the direct or reflex causation of the phenomena, the presence of acute rheumatism, and of various forms of heart disease, &c., &c. It is a matter, therefore, of ordinary experience that chorea minor will demand a treatment somewhat different from chorea major. The former, except under peculiarly unfavourable circumstances, has a natural tendency to get well of itself; but in saying this, it is only fair to say also that it takes a long time to do so. Rest is a most important element in treatment: not rest in bed in these numerous minor cases, useful as it is in the more severe ones; but rest from excitement, from noise, from harsh words, from taking part in the struggle of life. In these respects even the wards of an hospital are a great advance on the ordinary home of the city poor; but I am far from advocating hospital wards, with their sights and sounds, as the best place for chorea. A private room, with a bright, cheerful nurse, is far more desirable. Then, too, the quantity and nature of the food should be such as the nervous system requires, mainly fatty and farinaceous material, at regular intervals, and taken with due deliberation; the spasm of the facial muscles often leading to bolting the food without proper mastication.

In my own cases at the Bristol Royal Infirmary I used cold affusion in the majority of cases of chorea minor for many years. The mode of using this remedy is of great importance, as too rough-and-ready a method materially injures the patient. If the child is brought with gentleness and coaxing to the bath, and care is taken that there shall always be warm water to stand in, it will scarcely ever be frightened at the cold affusion. The pouring cold water over them should be done rapidly, but never so unexpectedly as to produce a shock. This remedy has always seemed to me to be beneficial, except in one instance, where the patient, who had lost her brother by drowning, could never be persuaded that she was not about to undergo the same fate.

I cannot speak with much personal experience of the use of electricity, as the nervousness of the little patients on the employment of this remedy has frequently seemed to render it more harmful than beneficial. But in many hands it has proved useful. Rosenthal prefers a continuous current, a stable current of moderate intensity being passed from the vertebral column to the nerves of the affected parts for three to five minutes. He has not been able to observe that the direction of the current exerts any influence upon the final result.

Charcot, however, states that he has found the ascending current increases choreic movements; the descending modifies them.

Perhaps one of the best methods for employing electricity is the one advocated by Dr. Dana in the *New York Medical Record.* He advises that a large sponge electrode of flexible brass (four by two inches) should be thoroughly moistened by salt water. The hair of the patient should also be thoroughly wetted. The electrode should be applied over the side of the head, above the ear, and on both sides if the chorea is bilateral. The other electrode should be placed in the hand of the affected side. The electrode on the scalp is made positive. A stable current of three to six cells should be used.

Of all the therapeutical remedies given in chorea, most observers seem to agree in the value of the mineral salts. A certain proportion of choreic cases are marked by anæmia, though I do not remember having seen any quite chlorotic. Trousseau, however, speaks of chlorosis being common, and even of the chorea of pregnancy being due to the anæmia sometimes induced by that state. In the anæmic cases iron has often proved of signal benefit. Dr. Ringer says that in simple uncomplicated cases of chorea arsenic is far the best remedy. Its occasional non-success is sometimes owing to the undue smallness of the dose; and decided improvement often begins simultaneously with a freer administration of the medicine. When chorea has resisted smaller quantities, children may take four, five, or more minims of the solution. In one case, which rapidly improved, he quickly increased the dose, until the boy took twenty minims of liquor arsenicalis six times a day; and in another successful case the girl took fifty minims of the liquor daily.

Whether arsenic acts by increasing the power of the heart in moderate doses; whether in chorea, as in anæmia, it increases both the red and the white corpuscles, the exact opposite of its effect on the blood in health; whether its influence is primarily on the vascular system; or whether, as seems more likely, it acts directly as a nervine tonic, we are still in doubt. Nor, indeed, do we quite know how a so-called nervine tonic acts. Is it on the walls of the vessels, or on the nerve-cells themselves? I presume really on both, rendering the process of interchange by osmosis more facile.

It is a matter of observation that females require larger doses of arsenic than males. The marked preponderance of the female sex affected by chorea is also recognised. Much as may be said in praise of arsenic in chorea, it is probable that the experience of the profession may militate against Dr. Ringer's doses, both as being in most cases larger than necessary, and certainly as not being invariably well-borne.

Thirty years ago my old master, Dr. Bence Jones, came to the conclusion, after many observations, that all the salts of the metals, including even those of lead, hastened recovery from chorea. But pre-eminent above all—and in my opinion superior in some respects even to arsenic—are the salts of zinc. Many practitioners recommend the oxide and the valerianate. I should be inclined to prefer the sulphate, beginning, of course, with doses of one or two grains, and rapidly mounting up to ten or twelve grains or more, three or four times a day.

So far then the recognised treatment has been devoted to the general principle of improving impaired nutrition of the nervous centres. But certain complications may demand additional remedies; and of these complications perhaps insomnia is the most troublesome. For this large doses of morphia have been given, hypodermically and otherwise, and doubtless with good results. We have, however, to remember that the majority of our patients are young, and that opium is not a comfortable remedy to use much with them. Very often, too, good results can be obtained by the bromides, by chloral hydrate, by hyosciamus, by camphor.

The experience of a single individual does not go for much; but in chorea minor I have not seen much advantage from chloroform. It has seemed to me in chorea, as in tetanus, to do absolutely nothing, beyond giving the patient a short sleep. This advantage, however, may have its value on occasion.

As to Calabar bean, which has been useful in Ogle's hands, one would view its administration with grave caution. The effects on the spinal cord are paretic. Apart from the paralysis sometimes the concomitant of chorea, apart from the muscular weakness so frequently observed, it is difficult not to feel that tremor, spasm, convulsion, incoordination, ataxy, contracture, and paralysis are very closely allied, and all form one family group. From this point of view Calabar bean might be contra-indicated. But a grain of experience is worth an ounce of theory, and any observation made by Ogle commands respect. In chorea, my own experience of Calabar bean is almost nil.

Acting on Dickenson's observation of dilatation of vessels, ergot has been given, and with good results.

A vast amount of benefit may be derived from a system of harmonised movements. The marching to a tune, the modified gymnastic exercises, never too prolonged, such as is obtained in many infant schools, seem gradually to, so to speak, teach co-ordination. I remember being much impressed many years ago with Dr. Güggenbuhl's system at his institution on the Abendburg for the treatment of cretins. A large number of these patients, on admission, were afflicted with incoordination of various movements, and one of his first efforts was directed towards bringing these movements into harmonious action. Every movement, therefore, in these early classes was made to the beat of a drum, and generally with the best results. Movement to music is, I think, one of our best weapons in chorea minor, especially if chronic, and is not without its use in the congenital form of the disease, and in the severer varieties of chorea major.

---

The treatment of chorea major is, however, not so straightforward. We have to deal not only with a more incessant and more violent jactitation, but almost invariably with some temporary mental weakness of the nature of imbecility, and with considerable incoordination of mastication, of swallowing, and of protrusion of the tongue. We have to trust to the resources of our art for giving us, in the treatment of this variety, that which we need so much in all diseases, the element of time. We have to consider by what portals death is attempting to enter, and to vary our remedies accordingly. Such patients run a risk from the condition of the heart, from cerebral rheumatism, from bed-sores and the fever consequent upon them, and still more from general nervous exhaustion.

To give time for restoring the impaired nutrition by food and nervine tonics, it is essential to prevent the system from being so rapidly worn out; and for this purpose agents must be had recourse to that we should shrink from using in the milder varieties of chorea.

Personally I have no experience of tartar emetic in my own cases, although I have seen it used largely in London in student days. Trousseau speaks well of it on the recorded results of earlier physicians, and its advantages in the treatment of delirium tremens would bear out the idea. It was given, I presume, to relieve cerebral hyperæmia. As in so many cases we have to deal with an anæmic badly nourished brain, its exhibition would seem frequently to be contra-indicated; and, apart from its depressing effects on the circulation, it is often difficult enough to feed our patients without artificially adding constant nausea to our perplexities. The digestion, too, is frequently already disturbed in this disease. Drs. Bonfils and Gillette have, however, expressed themselves in its favour; but in analysing their results they do not seem to have obtained rapid cures except in those instances of a second or third recurrence of the chorea, conditions which are always more easy to treat than the first attack. These French physicians have a special mode of administering this drug. They give it in gradually augmented doses for three days, and then they allow an interval of four or five days before a fresh series of doses is entered upon: this alternation of antimony and repose persisting until the symptoms disappear.

Various sedatives may have to be used with a free hand. Opium and chloroform inhalation may be as useful in this form of the disease as they are generally unnecessary in the milder varieties. Cannabis indica, so strongly recommended by Corrigan, is a weapon not to be despised; and conium is beneficial.

And when the motor phenomena are peculiarly violent, and we fear not only great exhaustion, but the formation of general bed-sores, the process of waddling, as explained by Trousseau, has certain advantages. The carefully surrounding each limb with cotton wool, and then, with full allowance for free respiration, the binding the waddled limbs to the trunk, seems to modify some of the dangers of the disease. I can speak well of it in two or three instances. But almost every remedy must yield in importance to constant feeding. The advantages of this feeding in the Weir Michell system for treating hysteria, the immense benefits derived from it in the cure of mental diseases, will be equalled, and even surpassed, in the morbid condition we are now considering.

Seclusion is very specially necessary. The freedom from even the least excitement is absolutely requisite; and therefore in this variety the wards of a hospital are contra-indicated.

As recovery progresses, much of the treatment recommended in the milder forms will be beneficial, and especially open-air exercise; but cold affusion is badly borne by the graver cases generally, although prolonged warm baths are useful.

It would not be right to pass over the use of strychnia, largely administered and lauded by so distinguished a physician as Trousseau. In his hands, and in gradually augmented doses that sound exceedingly large, it seems to have been useful. I have never had courage to push it to the extent he advises; and in smaller doses I have not thought it beneficial.

May I add one word, in which all the senior members will agree? One remedy may be useful for a series of years, and then may fail. Medical constitutions vary, more or less, in cycles of years. They have been proved to thus vary in France with reference to chorea; and in the exhaustive paper published by Dr. Strange, of Worcester, he relates how useful for a term of years he found wine in chorea, and how inert afterwards. This view opens up a series of points not included in our discussion to-night, but waiting for light from these very labours of the Investigation Committee. The influence of medical constitutions on treatment will have to be unravelled by a consideration of the disease on broad lines in this and other countries, with reference to atmospheric conditions, to soil, to family neuroses, to hereditary disease in general, and to the habits of the district and even of the nation.

